# Placental Pathology in Pregnancies with Maternally Perceived Decreased Fetal Movement - A Population-Based Nested Case-Cohort Study

**DOI:** 10.1371/journal.pone.0039259

**Published:** 2012-06-19

**Authors:** Brita Askeland Winje, Borghild Roald, Nina Petrov Kristensen, J. Frederik Frøen

**Affiliations:** 1 Division of Epidemiology, Norwegian Institute of Public Health, Oslo, Norway; 2 Center for Pediatric and Pregnancy Related Pathology, Department of Pathology, Oslo University Hospital Ullevål, Oslo, Norway; 3 Faculty of Medicine, University of Oslo, Norway; 4 Department of Pathology, Østfold Hospital Trust, Fredrikstad, Norway; Hospital Clinic, University of Barcelona, Spain

## Abstract

**Background:**

Decreased fetal movements (DFM) are associated with fetal growth restriction and stillbirth, presumably linked through an underlying placental dysfunction. Yet, the role of placental pathology has received limited attention in DFM studies. Our main objective was to explore whether maternal perceptions of DFM were associated with placental pathology in pregnancies recruited from a low-risk total population.

**Methods/Principal Findings:**

Placentas from 129 DFM and 191 non-DFM pregnancies were examined according to standardized macro- and microscopic protocols. DFM was defined as any maternal complaint of DFM leading to a hospital examination. Morphological findings were timed and graded according to their estimated onset and clinical importance, and classified in line with a newly constructed Norwegian classification system for reporting placental pathology. With our population-based approach we were unable to link DFM to an overall measure of all forms of placental pathology (OR 1.3, 95% CI 0.8–2.2, p = 0.249). However, placental pathology leading to imminent delivery could be a competing risk for DFM, making separate subgroup analyses more appropriate. Our study suggests a link between DFM and macroscopic placental pathology related to maternal, uteroplacental vessels, i.e. infarctions, placental lesions (intraplacental hematomas) and abruptions. Although not statistically significant separately, a compound measure showed a significant association with DFM (OR 2.4, 95%CI 1.1–5.0, p = 0.023). This association was strengthened when we accounted for relevant temporal aspects. More subtle microscopic materno-placental ischemic changes outside the areas of localized pathology showed no association with DFM (OR 0.5, 95%CI 0.2–1.4, p = 0.203). There was a strong association between placental pathology and neonatal complications (OR 2.9, 95% CI 1.6–5.1, p<0.001).

**Conclusions:**

In our population-based study we were generally unable to link maternally perceived DFM to placental pathology. Some associations were seen for subgroups.

## Introduction

Fetal growth restriction (FGR) is associated with significant risk for severe disabilities and death [1]–[3]. Risk can be reduced by appropriate assessment, but the ability to detect FGR in antenatal care remains weak [4]. The mother’s perception of fetal movement (FM) is still the simplest source of information about the baby’s well-being and should not be underrated. A maternal perception of decreased fetal movement (DFM) is widely reported to be associated with FGR [5]–[11]. DFM is also reported the days preceding an unexplained stillbirth [5], [12]–[14], suggesting that interventions could have prevented morbidity and mortality [15], [16]. The majority of women examined for perceived DFM in third trimester, however, continues with uncomplicated pregnancies [17]. So even if a maternal perception of DFM is rightly recognized as a good indicator of fetal compromise, its predictive value is low.

The well-documented association between DFM, FGR and stillbirth [1]–[4] is presumably linked to an underlying placental dysfunction [18]. Pathological processes in the placenta may lead to fetal hypoxia [19], either following profound acute circulatory insults such as abruptions and hemorrhages, or longstanding processes resulting in prolonged chronic hypoxia. When exposed to nutrient and oxygen restriction, it is hypothesized that the fetus will redistribute blood to vital organs [20] and will reduce non-vital activities such as gross fetal movements, [21]–[23]. Studies have reported that growth restricted fetuses have reduced fetal movement compared to controls [24] and that they demonstrate an almost dose-dependent reduction in FM during hypoxia [23], [25]–[27]. DFM has been found to be associated with abnormal placental morphology paralleling those seen in placentas in FGR pregnancies [18]. Although it is generally assumed and clinically plausible that DFM reflects fetal adjustment to a negative energy balance induced by reduced placental function, evidence to support this is limited. The first study on placental morphology was published just recently and reported altered placental structure and function with DFM [28], [29].

A perception of DFM often causes anxiety [30], [31] and results in frequent unscheduled third trimester antenatal visits [5], [8], [9], [16]. So far, however, placental pathology in DFM studies have been inadequately pursued [18]. A prospective FM counting study with a subsequent blinded study of the placenta was initiated to reveal information that may help to identify the DFM pregnancies at greatest risk.

The placenta substudy forms the basis for this report. Our main objective was to explore whether maternal perceptions of DFM were associated with placental pathology in pregnancies recruited from a low-risk total population. We hypothesized that DFM placentas would show morphological changes consistent with reduced placental function.

## Methods

### Ethics Statement

Written informed consent was obtained from all participants, both for the FM counting study and the morphological examination of the placenta following delivery. The study was approved by the Regional Committee for Medical Research Ethics, S-08694d, 2008/18353, 06.26.2009. There were no minors or legally incompetent participants in the study.

### The FM Counting Study

The placenta study is a case-cohort nested within a broader prospective FM counting study initiated to explore FM counting patterns and their relation to adverse pregnancy outcome. Thus all pregnancies included in this placenta study were selected among women who were already included in the population-based FM counting study.

From July 2009 to July 2011, all women with singleton pregnancies attending Østfold Hospital Trust for routine ultrasound screening in pregnancy week 17–19 were invited to the study. After written informed consent, a total of 2468 women were enrolled in the FM counting study, representing 42% of the eligible population. Among them, 1445 (59%) later submitted their FM chart and thus form the study group. Compared to the total population of pregnant women at Østfold Hospital Trust (data from Medical Birth Registry of Norway, year 2009 used as a reference [32]), the study group included more primiparous women (RR 1.2, 95%CI 1.2–1.3, p<0.001), fewer smoking mothers (RR 0.5, 95%CI 0.4–0.5, p<0.001), fewer cesarean sections (RR 0.8, 95%CI 0.7–0.8, p<0.001), fewer preterm (RR 0.8, 95%CI 0.6–0.9, p = 0.028), and low birth weight babies (RR 0.6, 95%CI 0.4–0.9, p = 0.006) (data not shown).

Participating women systematically recorded FM daily with a modified “count-to-ten=" approach, i.e. the time needed to perceive ten movements. The counting protocol was according to guidelines from the international collaboration Fetal Movement Intervention Assessment (FEMINA) [5], [8], [9], [33]. The information provided to women about DFM and when to seek medical attention is presented in full in Textbox S1 (Other 1). Women were not provided with any fixed limits for DFM, but advised to report significant and sustained decreases in the baby’s normal activity. In the current report DFM is defined as any maternal concern leading to a hospital examination.

### The Placenta Substudy

**Figure 1:**
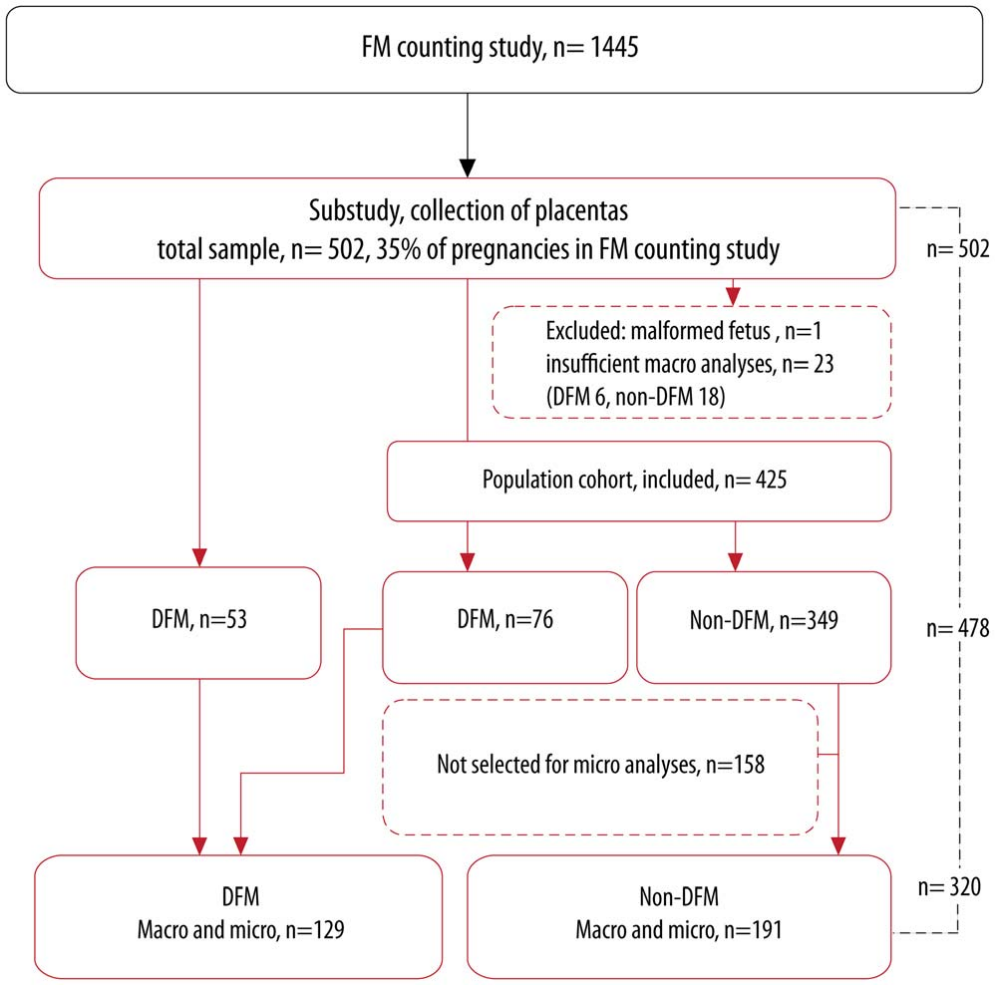
Flowchart for data collection.

From this prospective FM counting cohort there were two different criteria for eligibility to the placenta study: (i) if the mother had been examined in hospital care for a concern for DFM after 24^0^pregnancy week, or (ii) if the mother was among pregnancies preselected to the placenta study at time of enrollment in the FM counting study, independent of pregnancy outcome (a population-based sample as controls). Some of the women preselected to the population sample also experienced DFM and were included as DFM pregnancies in the analyses, Figure 1. Only babies without malformations were included in the analyses.

Since the placenta study was complementary to the FM counting study, a preceding power calculation for a case-control design was not performed. However, with our placenta sample a power calculation shows that we would have been able to detect an odds ratio for placental pathology of 2.0 in DFM compared to non-DFM pregnancies.

### Placental Examination

Information on gestational age, birth weight, and Apgar scores was available for pathologists at time of examination. All placentas were examined macroscopically by four designated pathologists according to a standardized protocol. All DFM placentas and a selection of non-DFM placentas were examined microscopically by a single, experienced pathologist with special interest in placental pathology (BR). The non-DFM placentas as controls were selected independently from their birth outcome. For every two DFM placentas we selected three non-DFM placentas (case-control ratio1∶1.5). The pathologist performing the microscopic examinations was blinded for macroscopic findings and DFM information.

Placentas were weighed (without cord and membranes), measured and inspected for focal lesions. Focal lesions were reported as estimated % of total placental volume, location central or peripheral and arbitrarily timed as acute (hemorrhagic changes) (<48 hours), subacute (hemorrhagic and fibrous changes) (2–20 days), or longstanding (fibrous changes) (> = 21 days). The gross macroscopic pathology was graded according to assumed clinical impact as:

1. **no pathology**: placentas without abnormalities
2. **minor impact**: abnormalities in placental shape, bilobate placenta, circumvallate placenta without bleeding, meconium stained membranes, peripheral infarctions (<10%)
3. **potential impact**: velamentous or marginal cord insertion, true knots
4. **moderate impact**: infarctions (central infarctions 5–9% or peripheral infarctions ≥10%)
5. **significant impact**: focal lesions like central infarctions and hemorrhages ≥10%, abruptions.

Standard tissue sections were taken from (i) membranes and umbilical cord, (ii) cord insertion site and placental near cord, (iii) full thickness macroscopically normal placenta, and (iv) two sections from the maternal plate. Additional sections were taken from centrally located focal parenchymal lesions. The sections were routinely formalin fixed, processed and embedded in paraffin. For the microscopic review only sections stained with Hematoxylin and Eosin (HE) were used.

Placental pathology from the microscopic examinations was categorized into nine groups according to a new Norwegian classification scheme [34], Table 1. The assumed clinical impact of the various processes was graded similarly to the macroscopic examination: (0) no pathology, (1) minor-, (2) potential- (3) moderate- and (4) significant impact, and timed accordingly. Only pathologies with clinical impact grade 3 and 4 are included as pathology in the analyses. Separate analyses are presented for pathologies with clinical impact grade 2 (potential impact). Analyses are based on the last DFM consultation if several.

**Table 1:** A systematic and standardized classification of placental pathology [34].

Category	Diagnostic categories
1	Normal placenta
2	Placenta with chorioamnionitis
3	Placenta with villitis (usually VUE)
4	Placenta with materno-placental circulatory disorder
5	Placenta with feto-placental circulatory disorder
6	Placenta with maturation disturbance
7	Placenta with findings suggestive of gene aberration
8	Placenta with placentation defect
9	Placenta with other pathology

Linking placental pathology present at term to early third trimester DFM consultations may be dubious. To get a more valid estimate of the association between DFM and placental pathology, we performed two separate subanalyses. First, we delimited the subset to DFM consultations occurring within the last seven days before birth and compared placental pathology between DFM and non-DFM pregnancies. With this approach the placental pathology would most likely precede the DFM consultation in time. Second, we delimited the subset to DFM consultations occurring within the last 21 days before birth and included *only* acute and subacute placental pathology, i.e. with estimated onset within the last 21 days, and compared these pathologies between DFM and non-DFM pregnancies. We were then able to assess whether DFM and placental pathology likely coincided in time.

Demographic indicators and information on birth outcome were collected from antenatal pregnancy charts and hospital records. Birth weight was adjusted for gestational age and sex. Baby weight below the 10^th^ percentile was classified as small for gestational age (SGA) [35], [36]. We have defined neonatal complications as preterm birth, SGA, infections, Apgar scores <7_5min_, or transfer to neonatal care unit for conditions relevant to fetal growth restriction or fetal distress, including respiratory syndrome and cerebral irritation. Classifications comply with definitions from Medical Birth Registry of Norway [32]. Respiratory distress is defined as typical signs or X ray findings, and cerebral irritation is defined as unrest, trembling, stiffness, and other signs of cerebral excitation [32].

### Statistical Analyses

We used SPSS version 17.0 (SPSS Inc., Chicago, IL, USA). To compare the likelihood of events between groups, we calculated odds ratios (OR) with 95% confidence intervals (CI) or relative risks (RR) when appropriate. Two samples *t* test was used to explore relationships between continuous variables. The level of statistical significance was set at p<0.05.

## Results

The final sample of 320 placentas with complete macroscopic and microscopic examinations consisted of 129 DFM and 191 non-DFM placentas. All babies included in the analyses were live born. For data collection, see flow chart Figure 1. There were no statistically significant differences in maternal characteristics between DFM and non-DFM pregnancies, Table 2.

**Table 2:** Maternal and fetal characteristics and birth outcome for DFM versus non-DFM pregnancies, from pregnancy week 24^0^ (n = 320).

	DFM pregnancies,n = 129	Non-DFM pregnancies,n = 191		
	n (%)	n (%)	OR (95% CI)	*p* ¶
**MATERNAL CHARACTERISTICS**				
Maternal age ≥35 yrs	19 (14.7)	34 (17.8)	0.8 (0.4–1.5)	0.469
Primiparous	79 (61.2)	108 (56.5)	1.2 (0.8–1.9)	0.403
Maternal obesity (Body Mass Index ≥30 kg/m^2^)	16 (12.4)	34 (17.8)	0.7 (0.3–1.2)	0.185
Maternal smoking in pregnancy	11 (8.5)	17 (8.9)	1.0 (0.4–2.1)	0.924
Pre-pregnancy maternal health or obstetric risk factors^a^	17 (13.2)	14 (7.3)	1.9 (0.9–4.1)	0.087
**DELIVERY ONSET**				
Spontaneous	91 (70.5)	143 (74.9)	0.8 (0.5–1.3)	0.392
Induced	28 (21.7)	40 (20.9)	1.0 (0.6–1.8)	0.581
Elective cesarean section	6 (4.7)	7 (3.7)	1.3 (0.4–3.9)	0.843
Emergency cesarean section (ECS) prior to contractions	4 (3.1)	1 (0,5)	6.1 (0.7–55.0)	0.108
**DELIVERY COMPLICATIONS**				
Intrapartum ECS on non-reassuring fetal state^b^	5 (3.9)	12 (6.3)	0.6 (0.2–1.8)	0.351
**FETAL CHARACTERISTICS AND BIRTH OUTCOME**				
Gestational age in weeks at birth, mean [range]	39^6^ [30^6^–42^5^]	39^6^ [30^3^–42^4^]	-	0.997
Birth weight in grams, mean [SD]	3568 (593)	3555 (506)	-	0.831
**Neonatal complications** l	26 (20.2)	37 (19.4)	1.1 (0.6–1.8)	0.863
Small for gestational age <10^th^ centile^ll^	14 (10.9)	21 (11.0)	1.0 (0.5–2.0)	0.968
Preterm birth (week 24^0^–36^6^)	8 (6.2)	7 (3.7)	1.7 (0.6–4.9)	0.298

¶ p-values refer to odds ratios for categorical data and t-test for continuous variables for comparisons between DFM vs non-DFM pregnancies.

a Maternal general health risk factors include: diabetes type I and II, chronic renal, hypertensive or coronary disease, inflammatory and collagen disease, epilepsy or coagulopathy. Obstetric risk factors include: previous pregnancy with FGR, stillbirth>21 weeks, fetal malformations, serious pre eclampsia, preterm delivery or spontaneous abortions >3.

b Non-reassuring fetal state: pathological CTG or Doppler or other signs of fetal distress.

l Neonatal complications: preterm birth, SGA, infections, Apgar scores <7_5min_ or transfer to NCU for conditions relevant to fetal growth restriction or fetal distress (respiratory syndrome or cerebral irritation).

ll Small for gestational age (SGA): birth weight for gestational age below 10^th^ percentile adjusted for maternal height and pre pregnancy weight and infant sex.

Generally, we were unable to link DFM to an overall measure of all forms of placental pathology with statistical significance (OR 1.3, 95% CI 0.8–2.2, p = 0.249), Table 3. However, DFM seems more closely associated with macroscopic placental pathology related to maternal, uteroplacental vessels, i.e. infarctions, placental lesions (intraplacental hematomas) and abruptions. All odds ratios for these subcategories were higher than unity, although not statistically significant separately. Yet, when these subcategories were merged, the compound measure showed a significant association with DFM (OR 2.4, 95%CI 1.1–5.0, p = 0.023), Table 3. This association was strengthened when we restricted the analysis to DFM consultations within the last seven days before birth (OR 3.0, 95%CI 1.1–7.6, p = 0.025). The same applied to the subsample with DFM consultations and estimated onset of placental pathology within the last 21 days preceding birth (OR 3.5. 95%CI 1.1–11.3, p = 0.038).

**Table 3:** Placental pathology in DFM pregnancies versus non-DFM pregnancies (n = 320).

		*Total DFM sample from pregnancy week 24*					*Subsample: DFM within the last 7 days before birth*			*Subsample: DFM within the last 21 days before birth and acute or subacute placental pathology (<21 days)*			
Characteristics		DFMpregnanciesn = 129	Non-DFMpregnanciesn = 191				DFMPregnanciesn = 45	*Comparison* *with* *non-DFM* *pregnancies* *n = 191*		DFMpregnanciesn = 82	*Comparison with* *non-DFM pregnancies* *with acute or subacute* *placental pathology* *(<21 days) n = 191*		
		n (%)	n (%)		OR (95% CI)	*P* ¶	n (%)	OR (95% CI)	*P* ¶	n (%)	OR (95% CI)		*P* ¶
**PLACENTA CHARACTERISTICS**													
Trimmed weight in grams, mean [range]		575 [274–1000]	584 [310–1010]		-	0.571	580 [274–915]		0.871				
Fetal Placental weight ratio		6.4 [1.3]	6.3 [1.2]		-	0.436	6.2 [1.4]		0.774				
**CORD ANOMALIES**		7 (5.4)	13 (6.8)		0.8 (0.3–2.0)	0.618	4 (8.9)	1.3 (0.4–4.3)	0.628				
True umbilical cord knot		3 (2.4)	4 (2.1)				0						
Velamentous insertion site		2 (8.3)	3 (7.0)				2 (4.4)						
Marginal insertion site		2 (8.3)	7 (16.3)				2 (4.4)						
**PLACENTAL PATHOLOGY**													
ANY PATHOLOGY^a^	40 (31.0)			48 (25.1)	1.3 (0.8–2.2)	0.249	12 (26.7)						
*INFECTIONS*	11 (8.5)			13 (6.8)	1.3 (0.6–2.9)	0.567	2 (4.4)						
Chorioamnionitis	8 (6.2)			12 (6.3)			1 (2.2)						
Villitis	3 (2.3)			1 (0.5)			1 (2.2)						
*MATERNO-PLACENTAL CIRCULATORY DISORDERS, TOTAL*	25 (19.4)			29 (15.2)	1.3 (0.8–2.4)	0.327	9 (20.0)	1.4 (0.6–3.2)	0.431	8 (9.8)	16 (8.4)	1.2 (0.5–2.9)	0.712
**Materno-placental insufficiency, abrupt circulatory insults**	19 (14.7)			13 (6.8)	2.4 (1.1–5.0)	0.023	8 (17.8)	3.0 (1.1–7.6)	0.025	7 (8.5)	5 (2.6)	3.5 (1.1–11.3)	0.038
Infarctions	15 (11.6)			13 (6.8)	1.8 (0.8–3.9)	0.139	7 (15.6)			4 (4.9)	5 (2.6)		
Central infarctions > = 5%	10 (7.8)			10 (5.2)	1.5 (0.6–3.8)	0.365	6 (13.3)			3 (3.7)	4 (2.1)		
Peripheral infarctions > = 10%	5 (3.8)			3 (1.6)	2.5 (0.6–10.8)	0.210	1 (2.2)			1 (0.5)	1 (1.2)		
Placental lesions	2 (1.6)			0			0			2 (2.4)	0		
Abruptions/hemorrhages	4 (3.1)			1 (0.5)			2 (4.4)			3 (3.7)	0		
**Materno-placental insufficiency, ischemic changes**	6 (4.7)			16 (8.4)	0.5 (0.2–1.4)	0.203	1 (2.2)	0.2 (0.0–1.9)	0.183	1 (1.2)	11 (5.8)	0.2 (0.0–1.6)	0.129
*MATURATION DISORDERS* b	2 (1.6)			4 (2.1)	0.7 (0.1–4.0)	0.726	2 (4.4)						
*CORD PATHOLOGY* c	6 (4.7)			8 (4.2)	1.1 (0.4–3.3)	0.843	1 (2.2)						

¶ p-values refer to odds ratio for categorical data and t-test for continuous variables for comparisons between DFM vs non-DFM pregnancies.

a Include all cases with pathology with assumed moderate to important clinical impact from macroscopic or microscopic examination.

b Maturation disorders, pathology was not timed.

c Cord pathology includes cases with uncoiled umbilical cord (n = 8), single umbilical artery (n = 3), thrombosis (n = 2), severe edema (n = 1), cord pathology was not timed.

There was no association with DFM for more subtle microscopic materno-placental ischemic changes outside the areas of localized pathology (OR 0.5, 95%CI 0.2–1.4, p = 0.203), Table 3. In cases of acute chorioamnionitis we found no association, as the DFM consultations preceded the pathology onset by large margins and thus were unrelated. Placentas from DFM and non-DFM pregnancies were similar in mean trimmed weight and mean fetal placental weight ratio across samples. A quintile distribution of placental weight showed no differences between DFM and non-DFM pregnancies. Fetal vessels in the membranes are vulnerable to injury and thrombosis, and are more susceptible to compression by fetal parts resulting in obstruction of blood flow. We found cord anomalies, including true knots and velamentous and marginal cord insertions, to be similar between the groups, Table 3. Since abnormal cord insertion site has been linked to SGA [37] as well as DFM [29], we restricted the analysis to include only velamentous and marginal cord insertions. However, results were the same (data not shown).

Ten women had more than one DFM consultation. This is presented in Table 4 with information on gestational age at time of DFM and days between DFM and birth. We found no association between having recurrent DFM consultations and overall placental pathology (OR 1.5, 95% CI 0.4–5.8, p = 0.525) or between having recurrent DFM consultations and neonatal complications (OR 1.9, 95% CI 0.8–4.6, p = 0.167). For DFM infants later diagnosed as SGA, the median time between diagnosis of intrauterine growth restriction (fetal weight estimate <–10% by ultrasound measurement) and delivery was 20 days, range 2–63.

**Table 4:** Characteristics of consultations for DFM from 129 pregnancies.

CHARACTERISTICS OF DFM CONSULTATIONS	First DFM consultation, n = 129	Second DFM consultation, n = 10	Third DFM consultation,n = 2
	Median [range]	Median [range]	Median [range]
Gestational age in weeks at time of DFM consultation	37.3 [24.1–41.5]	37.7 [31.6–40.7]	38.6 [36.7–40.6]
Days between DFM consultation and delivery	14 [0–122]	13 [1–46]	3.5 [2]–[5]

On birth outcome a strong association between placental pathology and neonatal complications was found, Table 5. The strongest associations with birth outcome were seen for placental pathology in category four according to the Norwegian classification system, i.e. materno-placental circulatory disorders. This category relates to maternal vascular pathology. It includes both longstanding, chronic placental processes such as old infarctions and diffuse ischemic changes, and acute episodes that have occurred closer to birth like abruptions. These associations were present both for the more abrupt circulatory insults and the subtle ischemic changes.

Neither placental ascending infections (placental pathology category two) nor cord anomalies were associated with the birth outcomes, Table 5. The remaining categories were small, which limited subgroup analyses.

**Table 5:** Placental pathology by birth outcome (n = 320).

Characteristics	Placental pathology	No placental pathology		
	n (%)	n (%)	OR (95% CI)	*P* ¶
**PLACENTA CHARACTERISTICS**				
Trimmed placental weight in grams, mean [range]	546 [274–1000]	594 [286–1010]	-	0.011
**BIRTH OUTCOME**				
*PLACENTAL PATHOLOGY, TOTAL* a				
**Neonatal complications** l	29/88 (33.0)	34/232 (14.7)	2.9 (1.6–5.1)	<0.001
Small for gestational age <10^th^ centile^ll^	19/88 (21.6)	16/232 (6.9)	3.7 (1.8–7.6)	<0.001
Preterm birth (week 24^0^–36^6^)	4/88 (4.5)	11/232 (4.7)	1.0 (0.3–3.1)	0.941
*MATERNO-PLACENTAL CIRCULATORY DISORDERS, abrupt circulatory insults*				
**Neonatal complications**	10/32 (31.3)	53/288(18.4)	2.0 (0.9–4.5)	0.088
Small for gestational age <10^th^ centile	9/32 (28.1)	26/288(9.0)	3.9 (1.6–9.4)	0.002
Preterm birth (week 24^0^–36^6^)	2/32 (6.3)	13/288 (4.5)	1.4 (0.3–6.6)	0.661
*MATERNO-PLACENTAL CIRCULATORY DISORDERS, ischemic changes*				
**Neonatal complications**	11/22 (50.0)	52/298 (17.4)	4.0 (1.9–11.5)	0.001
Small for gestational age <10^th^ centile	7/22 (31.8)	28/298 (9.4)	4.5 (1.7–12.0)	0.003
Preterm birth (week 24^0^–36^6^)	2/22 (9.1)	13/298 (4.4)	2.2 (0.5–10.4)	0.323
*CORD ANOMALIES WITH POTENTIAL IMPACT* §				
**Neonatal complications**	4/20 (20.0)	59/300 (19.7)	1.0 (0.3–3.2)	0.971
Small for gestational age <10^th^ centile	3/20 (15.0)	32/300 (10.7)	1.5 (0.4–5.3)	0.550
Preterm birth (week 24^0^–36^6^)	1/20 (5.0)	14/300 (4.7)	1.1 (0.1–8.6)	0.946

¶ p-values refer to odds ratio for categorical data and t-test for continuous variables for comparisons between pregnancies with or without placental pathology.

a Includes all cases with pathology with assumed moderate to important clinical impact from macroscopic or microscopic examination.

l Neonatal complications: preterm birth, SGA, infections, Apgar scores <7_5min_ or transfer to NCU for conditions relevant to fetal growth restriction or fetal distress (respiratory syndrome or cerebral irritation).

ii Small for gestational age: birth weight for gestational below 10^th^ percentile adjusted for maternal height and pre pregnancy weight and infant sex.

§ Includes the cord anomalies; true umbilical cord knots (n = 7), velamentous (n = 5) and marginal cord (n = 9) insertion.

We found no statistically significant differences in birth outcome between DFM and non-DFM pregnancies, Table 2.

### Representativeness of Sample

Placentas from approximately two thirds of the DFM pregnancies were eventually included: 85% from DFM pregnancies that were among pregnancies initially preselected to the population sample and 46% from DFM pregnancies outside the population sample, Figure 1. In 78 (38%) of the DFM pregnancies the placenta was lost to the study. A sensitivity analysis showed that DFM pregnancies with (n = 129) or without (n = 78) the placenta included were similar in terms of mean infant birth weight (3555 grams (SD 609) versus 3634 grams (SD 523), p = 0.350) and mean gestational age at birth (40^0^ weeks (SD 2) versus 40^3^ weeks (SD 1.6), p = 0.156). They were also similar in terms of neonatal complications (OR 1.1, 95% CI 0.5–2.1, p = 0.872), SGA (OR 0.9, 95% CI 0.4–2.3, p = 0.854) and preterm infants (OR 1.2, 95% CI 0.4–4.2, p = 0.749).

## Discussion

To our knowledge, this is the first population-based study that compares placental morphology in DFM and non-DFM pregnancies. We were unable to link DFM to placental pathology with statistical significance, although our data suggest higher odds for a subgroup of placental pathology in DFM-pregnancies, primarily related to abrupt circulatory insults. The maternal ability to detect SGA and neonatal complications was limited. With our population-based approach we faced the well-known challenge of low power when studying rare events in prospective cohort designs. However, the placenta study is part of a broader FM counting study where this design was most suitable.

Generally, placental pathology contributed little to explain third trimester maternally perceived DFM. Two factors need to be mentioned. First, some forms of placental pathology are known to trigger imminent delivery, among them acute chorioamnionitis, and may serve as competing risk for DFM. An overall measure of placental pathology could therefore be misleading, since placental pathology relevant for DFM may be underestimated. So subgroup analyses may be more appropriate. Second, linking placental pathology present at term to early third trimester DFM consultations may be dubious. Placental pathology must precede the DFM consultation in time to be relevant for DFM. By necessity, a retrospective estimate of pathology onset is broad, especially for pathology with estimated onset >21 days prior to birth.

To get a more valid estimate of the associations between DFM and placental pathology, we therefore restricted the analyses to include only DFM consultations occurring within the last seven days before birth. The result from this subgroup analysis confirmed and even strengthened the result from the total sample; DFM seemed more associated with abrupt, major circulatory insults resulting from obstruction of maternal uteroplacental vessels. Discrepancies between the two analyses were primarily linked to cases of acute chorioamnionitis, which were unrelated in time with the DFM consultation, similar to what was seen for the more diffuse ischemic changes.

The same result emerged when we restricted the analysis to include DFM pregnancies occurring within the last 21 days before birth and compared *only* placental pathology with acute or subacute onset (<21 days) between DFM and non-DFM pregnancies, implying that events were more likely to have coincided in time. Again the associations between DFM and macroscopic maternal vascular pathology were strengthened. Thus associations between DFM and more abrupt circulatory events remained also when temporal associations were accounted for. The clinical implications are, however, not clear. Important macroscopic indicators of placental function, such as placental trimmed weight and fetal placental weight ratio, were not different between DFM and non-DFM pregnancies in our study, indicating overall healthy placentas with substantial reserve capacity in both groups. Placental weight has previously been found to be predictive of maternal disease, obstetric outcome and perinatal morbidity and mortality [38].

There are few studies to support or refute our findings, as research linking placental dysfunction to DFM is scarce. The first study actually investigating placenta morphology in DFM pregnancies was presented just recently [28], [29]. In comparing placentas from 36 DFM pregnancies with 36 healthy controls, striking differences were reported. Placentas from DFM pregnancies were smaller (lighter, with smaller surface area), had more macroscopic infarctions, and were more likely to have abnormal shape and eccentric cord insertion than those from healthy pregnancies. Microscopic examination revealed ischemic changes indicating maternal vascular pathology with increased number of syncytial knots, fewer blood vessels, and reduced area of trophoblast per villus [28], [29].

While these results apparently differ from our findings, direct comparison may be deceiving. The previous study included only pregnancies where perceived DFM lasted more than 12 hours and where the baby was delivered within seven days of presentation, representing 12% of the DFM consultations in the study (36/305). These selected high-risk DFM pregnancies were compared with selected healthy controls, i.e. sick versus healthy. In our population-based approach, we compared women with and without a maternal complaint for DFM without further selection, a measure known to have low predictive value, but important in clinical practice. These differences in design are clearly reflected in the study samples. Their DFM sample included a substantial number of smaller placentas with lower fetal placental weight ratio, whereas DFM and non-DFM placentas in our population-based sample were comparable in size. The differences in placental ischemic changes in the two studies may mainly reflect the differences in the study cohorts, partially also differences in criteria and classification. The pathology examination procedure differed between the studies. Again our study has a focus on every-day approaches. We have thus used standard, routine examination protocols, both in the macroscopic and microscopic examinations, assessing HE sections only.

While acknowledging that differences in our study were expected to be smaller, our design deliberately aimed at being relevant for the everyday situations facing obstetricians and midwives. With the similarities in aims between our and the previous study, we have purposely amended our analysis where appropriate to facilitate comparison. The studies are thus complementary and each provides building blocks to fill in the knowledge gaps.

The association between placental pathology and FGR and stillbirth has previously been documented [39]–[41]. Consistent with these studies we found strong associations between SGA and placental pathology, both for the non-macroscopic, microscopically identified ischemic changes and for the macroscopically more abrupt, circulatory insults. However, we only found an association with DFM for the latter category. Birth outcomes appear similar between DFM and non-DFM pregnancies. This is in line with the overall result of minor differences in placental pathology between DFM and non-DFM placentas in our study. In addition, the effect of focal obstruction of maternal uteroplacental vessels is potentially less severe in normally sized placentas, as in our sample, with capacity for compensatory mechanisms. Thus major differences in birth outcome should not be expected.

Other factors may also have improved birth outcomes for DFM pregnancies. The effect of being included in a study often influences participant behavior, usually in a beneficial direction [42], i.e. maternal care seeking behavior. Improved clinical care such as appropriate management, timing of delivery and delivery interventions may be more likely in DFM pregnancies. Our study was, however, neither designed for nor powered to measure such effects of maternal monitoring of FM on birth outcome.

The strong association between placental pathology and neonatal complications may have been reinforced as pathologists were informed about gestational age, birth weight and Apgar scores. This information is, however, vital in placental examination since placenta is a dynamic organ, constantly developing and maturing throughout pregnancy. In terms of DFM, a number of macro examinations were conducted during parts of the study period when DFM placentas were the only placentas collected, implying that pathologists were inevitably aware of DFM status during part of the study period. The strict protocol for macroscopic registration and standardized sectioning makes this awareness less prone to bias. Importantly, when micro examinations were conducted, the pathologist was unaware of DFM status. The well defined microscopic criteria of the newly constructed Norwegian classification scheme were strictly applied.

Consistent with earlier studies [33], [43], [44], we found that the predictive value of maternally perceived FM for identification of SGA and neonatal complications was low. Women’s perception of FM is known to be affected by pathological and non-pathological entities [44]. A valuable contribution from the previous study was that it managed to link DFM to placental pathology [29]. However, this was based on a highly selected risk group of DFM pregnancies representing less than one percent of its obstetric source population. With our population-based approach we were unable to replicate these results, illustrating how difficult it can be to interpret DFM both for the mothers and health care professionals. Since women will continue to report concerns for DFM [5], [8],[9],[16], simple tools to help mothers maintain a safe pregnancy is needed.

In a recent Lancet series on stillbirth prevention, screening for placental insufficiency and better management of DFM pregnancies were rated among top ten research priorities [15]. A natural first step would be to improve women’s ability to recognize the important changes in FM so as to ensure appropriate care-seeking behavior. In the broader FM counting study, placenta data will be included as an objective measure to explore whether FM counting patterns contain information that can support maternal common sense. Given appropriate care seeking behavior, the potential role of placental biomarkers may provide a promising supplement to identify those DFM pregnancies at highest risk of poor outcome. Preliminary results from a DFM study have reported that DFM pregnancies with poor birth outcome showed reduced plasma concentrations of hCG and hPL compared to DFM pregnancies with normal outcome [21], [41]. Dysregulation of placental function was suggested as a clue to the underlying pathology.

### Conclusion

In our population-based study we were generally unable to link maternally perceived DFM to underlying placental pathology, although some associations were seen for subgroups. Maternal ability to identify FGR was low. In order to enhance the role of FM counting, further research must focus on ways to help women to identify fetal compromise from chronic placental pathology.

## Supporting Information

Textbox S1 **Information about decreased fetal movement (DFM) provided to the mothers.**
(DOC)
