# Supplementary material for: Placental Pathology in Pregnancies with Maternally Perceived Decreased Fetal Movement - A Population-Based Nested Case-Cohort Study
Source: PLoS One. 2012 Jun 19;7(6):e39259. doi: 10.1371/journal.pone.0039259 (PMC3378526; doi:10.1371/journal.pone.0039259)
Supplement: Textbox S1 — Information about decreased fetal movement (DFM) provided to the mothers. (DOC) [file pone.0039259.s001.doc]

### Textbox 1. Information about decreased fetal movement (DFM) provided to the mothers

*If you are worried about your baby, regardless of reason, you should seek advice and help from your doctor or midwife. If you are concerned because your baby is less active as the weeks go by, you should bring the kick count form to your next pregnancy check-up.*

*In some cases you should contact the maternity unit directly:*

- *If your baby does not kick one day, you should never wait until the next day.*
- *If your baby kicks progressively less during the day/days and you feel decreased activity.*

*If you are in doubt about what is low activity, you should know it is rare that a healthy baby kicks less than 10 kicks within a two-hour period when you know the baby is usually active. If you feel the baby has kicked scarcely the whole day, you should contact the maternity unit. If you are unsure of your baby’s activity on a given day, before you started that day’s counting, you should be alert. Make sure you count again within the next 12 hours, and contact the maternity unit if the results are repeated.*
